# Does Artificial Intelligence Bring New Insights in Diagnosing Phlebological Diseases?—A Systematic Review

**DOI:** 10.3390/biomedicines13040776

**Published:** 2025-03-22

**Authors:** Sergiu-Ciprian Matei, Sorin Olariu, Ana-Maria Ungureanu, Daniel Malita, Flavia Medana Petrașcu

**Affiliations:** 1 Abdominal Surgery and Phlebology Research Center, “Victor Babeș” University of Medicine and Pharmacy, 300041 Timișoara, Romania; matei.sergiu@umft.ro (S.-C.M.); olariu.sorin@umft.ro (S.O.); 2 1st Surgical Department, Pius Brînzeu Emergency County Hospital, 300723 Timișoara, Romania; 3 Department XV, Clinic of Radiology and Medical Imaging, “Victor Babeș” University of Medicine and Pharmacy, 300041 Timișoara, Romania; malita.daniel@umft.ro; 4 Department of Radiology and Medical Imaging, “Pius Brînzeu” Emergency County Hospital, 300723 Timișoara, Romania; 5 Department of Biochemistry, “Victor Babeș” University of Medicine and Pharmacy, 300041 Timișoara, Romania; flavia.petrascu@umft.ro

**Keywords:** artificial intelligence, Doppler ultrasound, duplex scan, chronic venous disease, venous reflux, deep vein thrombosis

## Abstract

**Background/Objectives**: Artificial intelligence (AI) is rapidly transforming the landscape of modern medicine, offering advanced tools for diagnosing complex conditions. In the realm of venous pathologies such as chronic venous disease (CVD), venous reflux, and deep venous thrombosis (DVT), AI has shown tremendous potential to improve diagnostic accuracy, streamline workflows, and enhance clinical decision-making. This study aims to evaluate the efficacy and feasibility of AI algorithms in diagnosing venous diseases and explore their potential impact on clinical practice. **Methods**: This paper provides a comprehensive review of key studies documenting the use of AI in venous pathology diagnostics, with different electronic databases being searched, including MEDLINE/Pub Med, Web of Science, Scopus, Embase, ResearchGate, and Google Scholar. **Results**: Out of 52 reports assessed for eligibility, 43 were excluded according to the preset criteria; therefore, findings from nine major studies involving more than 1000 patients were analyzed. The evaluation shows that AI utilization in the diagnosis of venous pathologies has demonstrated significant improvements. Notably, AI algorithms have achieved an accuracy exceeding 90%, significantly reducing inter-observer variability and ensuring consistent interpretation of ultrasonographic images across different clinicians and settings. Additionally, AI has accelerated diagnostic workflows, decreasing the time required for image analysis by more than 50%. Furthermore, AI has proven capable of detecting subtle abnormalities, such as minor venous reflux or early-stage thrombi, which may be overlooked during manual evaluations. **Conclusions**: Artificial intelligence represents a transformative innovation in the diagnosis and management of venous diseases. By enhancing diagnostic accuracy, streamlining workflows, and enabling personalized care, AI has the potential to address current challenges in venous diagnostics and improve patient outcomes. The future of AI in venous diagnostics is promising, and several areas of development were noted, including AI algorithms embedding directly into ultrasound devices to provide instantaneous diagnostic insights during patient evaluations; combining AI-processed Doppler data with other imaging modalities, such as computed tomography or MRI, for comprehensive assessments; AI usage in order to predict disease progression and tailor treatment strategies based on individual patient profiles; and constructing large-scale, multicenter datasets to improve the robustness and generalizability of AI algorithms.

## 1. Introduction

Chronic venous disease (CVD) involves various pathological and hemodynamic changes which affect the veins of the lower limbs, leading to a broad spectrum of symptoms and clinical manifestations. It is highly prevalent in the general population and can result in significant disability in its more severe stages [1,2]. The spectrum of clinical manifestations for CVD is very diverse, including varicose veins, chronic venous insufficiency, stasis dermatitis, venous ulcers, superficial venous thrombosis, reticular veins, and telangiectasias [3,4].

Despite the fact that in most cases the diagnosis can be established in a clinical setting, ultrasound (US) scanning remains a valuable tool in venous evaluation [5,6], including diagnosis and preoperative mapping to identify adequate veins before surgery [7,8]. Also, ultrasound assessment of vein wall thickness could help to determine the energy level necessary in case of endovenous laser ablation, and the sclerosing agent concentration in case of chemical ablation [9].

US protocols include grayscale, color Doppler, and spectral Doppler. Recommended lower-extremity protocols include central leg and calf veins. Duplex Doppler is widely used to evaluate patients with chronic venous disease, especially with suspected venous reflux. US for thrombosis can be characterized as normal, acute DVT, superficial thrombosis, or chronic post-thrombotic changes in most patients [10]. Although imaging relies primarily on duplex ultrasonography, in selected cases it could be supplemented by computed tomography and magnetic resonance venography, and intraoperatively by intravascular ultrasonography [11]. Also, in advanced stages of CVD, additionally to ultrasound scanning [12], infrared thermography can be useful to prognoses of the venous leg ulcer healing process [13].

Overall, all the guidelines recommend lower-limb venous system US scanning in all patients presenting symptoms related to CVD [14].

Despite initial controversies, artificial intelligence (AI) is gaining more and more ground in various fields, including in medical practice [15]. Artificial intelligence (AI) models have proved to be promising tools, identifying predictive features related to medical records data. However, their use in everyday clinical practice is still a challenge, mainly due to technical limitations [16]. Nonetheless, AI algorithms, particularly deep learning, have demonstrated remarkable progress in image-recognition tasks [17], radiology and medical imaging being one of the main fields where AI has started to be used [18].

While US is expanding globally as a first-line imaging technique in various clinical fields, including vascular assessment, it is operator-dependent. As a result, trained physicians manually gather and visually assess images for disease detection, identification, and monitoring. However, the diagnostic accuracy is inherently limited due to the operator-dependent nature of ultrasound. In contrast, AI demonstrates exceptional ability in automatically identifying complex patterns and offering quantitative analysis of imaging data, showing great potential to enhance physicians’ accuracy and consistency in diagnostics [19].

Additionally, various other AI models have been explored in other fields of vascular medicine assessment, including vascularity of postoperative flap monitoring [20], or machine learning techniques usage in order to identify hidden patterns and complex associations in health data without any a priori assumptions, specifically focusing on aortic aneurysms, lower extremity arterial disease, and carotid stenosis [21]. Also, artificial intelligence can be used for risk stratification and treatment approaches individualization [22]. Machine learning models provided a good accuracy in predicting one-year outcomes following endovascular aortic repair procedures too, performing better than logistic regression [23].

The current study aims to present an up-to-date analysis of published data related to the use of AI to improve the diagnosis of venous diseases, as well further perspectives on integrating AI in daily practice.

## 2. Materials and Methods

### 2.1. Definition of Scope and Objective

As AI attracts more and more interest in medicine, the objective of our review was to assess all the current data regarding AI usage in lower-limb venous disease diagnosis. Thus, aiming to provide novel insights and to address aspects that have potentially remained underexplored in the academic discourse, some research questions (RQs) were formulated, as follows:

RQ: How does the existing literature capture AI involvement in venous disease diagnosis up to today? This objective aimed to provide a comprehensive understanding of how the current body of literature addresses the concept of AI usage in venous disease diagnosis, including disease type/stage, complications, and AI models used.

RQ: How accurate are the models provided by AI in venous disease diagnosis? Despite the overall enthusiasm for implementing AI in medicine, an objective analysis of the real potential of AI insights should be performed. This objective aimed to assess the data regarding accuracy, sensitivity, and specificity of AI algorithms in different situations, including clinical images and imagistic investigation analyses.

RQ: Should we adopt AI in current clinical practice? This objective aimed to analyze the feasibility, and as well the future directions and perspectives, of implementing AI in phlebology. By answering this question, we tried to deliver some insights to guide phlebologists and physicians in their considerations of this field.

### 2.2. Study Protocol and Literature Research

A comprehensive and extensive literature research was conducted across different electronic databases, including MEDLINE/Pub Med, Web of Science, Scopus, Embase, ResearchGate, and Google Scholar, in order to analyze the existing literature on AI’s impact on venous disease diagnosis. Literature published up until January 2025 was screened, in order to include the most current studies available on this key topic. The screening strategy employed a wide range of keywords and phrases relevant to this paper’s aims. Key search terms included “artificial intelligence”, “deep learning”, “machine learning”, “ultrasound”, “doppler”, “duplex”, “venous mapping”, “venous reflux”, “chronic venous disease”, “deep vein thrombosis”, and “diagnosis”. For a more precise search, Boolean operators (“AND”, “OR”, and “NOT”) were strategically employed to refine and link the search terms effectively. The search string was constructed as follows: (“artificial intelligence” [MeSH] OR “deep learning” OR “machine learning”) AND (“doppler ultrasound” [MeSH] OR “duplex scan” OR “venous mapping”) AND (“venous reflux” [MeSH] OR “chronic venous disease” OR “deep vein thrombosis”) AND (“diagnosis” [MeSH] OR “differential diagnosis” OR “assessment”).

In order to ensure a structured, transparent, and reproducible methodology, this paper was conducted according to PRISMA (Preferred Reporting Items for Systematic Reviews and Meta-Analyses) guidelines [24]. This paper has been registered with the Open Science Framework (OSF) with the registration code osf.io/yb9ev. No ethical approval was required for this study as this was a literature review.

**Figure 1:**
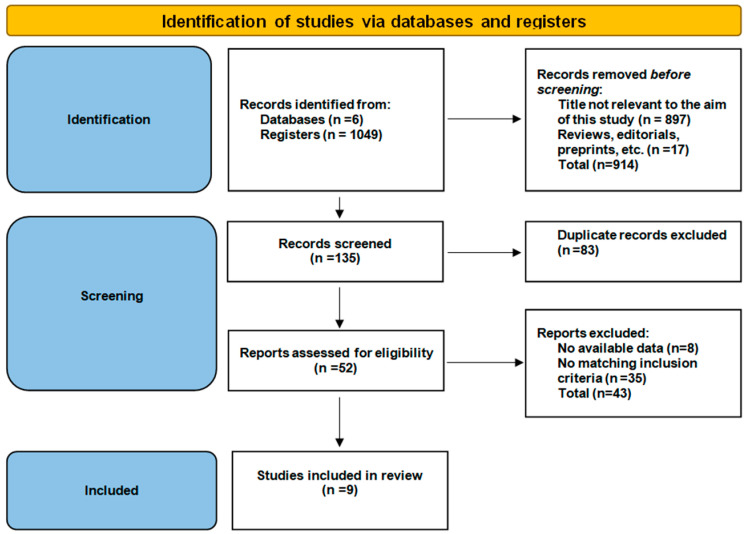
PRISMA flow diagram.

PRISMA flow diagram is presented in Figure 1.

### 2.3. Inclusion and Exclusion Criteria

The literature selection was conducted by employing explicit inclusion and exclusion criteria, in order to ensure transparency and scientific rigor, as well.

The following inclusion criteria were established:

- study methodology: studies must involve/analyze medical records from human subjects, including different types of imagistic materials (pathological results, ultrasound, magnetic resonance, photographic images, etc.);
- the abstract explicitly highlights the topic of AI involvement in assessing lower-limb veins;
- focus on venous disease diagnosis—research specifically needed to mention how AI was used in order to establish the diagnosis;
- only peer-reviewed articles published in English were included.

The following exclusion criteria were established:

- non-human studies/papers, including in vitro or technical models, were excluded in order to maintain the focus on clinical outcomes in human patients;
- lack of specific outcomes: in order to ensure the focus of our study on the clinical applicability of AI models in phlebology, papers which presented only experimental models or did not provide specific outcomes for the AI model used were not included.
- the content focuses only on AI impact on arterial, abdominal veins or other cardiovascular diseases diagnosis: in order to maintain the focus of our review on phlebology, mixed papers which presented different AI models used in both vein and artery assessment, or in other vascular disease assessment (including arteries, abdominal large vessels, etc.) were excluded.
- the abstract does not cover AI involvement in venous disease diagnosis: in order to ensure that the analysis is strictly within the scope of this research, papers whose abstracts exhibited a complete absence of any reference to AI involvement in venous diseases were excluded;
- full paper not accessible: articles that looked relevant from the title and first information, but were not accessible, were excluded from this analysis;
- non-English papers were excluded from our analysis due to limitations in the authors’ language proficiency;
- duplicate papers, preprints, reviews, commentaries, and editorials: in order to maintain the reliability and credibility of the data included and analyzed in this review, grey literature was excluded.

### 2.4. Risk of Bias and Quality Assessment

For the systematic assessment of study quality and determination of risk of bias within the included studies, our review employed a dual approach, integrating both qualitative and quantitative evaluation methods. Initially, the quality of observational studies was evaluated using GRADE [25]. To ensure the objectivity and reproducibility of our quality assessment process, each study was independently evaluated by two reviewers. Discrepancies in quality assessment scores were resolved through consultation with a third reviewer. When evaluating the efficacy and feasibility of AI in the diagnosis of venous pathologies using evidence-based medicine (EBM) as a reference framework [26], it is essential to acknowledge that research in this field is still in its early stages. The inclusion criterion for this review was a minimum Grade B level of evidence, which corresponds to moderate evidence derived from Level II–III studies. As research advances and more high-quality RCTs are conducted, Grade A evidence will be incorporated as the criterion to further strengthen the assessment of AI applications in venous disease diagnosis.

### 2.5. Data Collection Process

Out of 1049 candidate published papers which were initially searched, only 9 met the inclusion criteria, the topic being discussed to date. These 9 papers represented the main literature corpus of our review, 89% (n = 8) of them being published in the past 4 years.

## 3. Results

### 3.1. Key Studies on Artificial Intelligence Usage in Venous Pathology Diagnosis and Their Characteristics

This systematic review analyzed a total of 9 studies focused on AI involvement in venous diameter, venous reflux, or venous thrombosis assessment and diagnosis. The literature corpus of our review included papers published from December 2018 to April 2024. Considering the study designs, all were either prospective or observational studies (Shi Q et al. [27]; Levshinskii et al. [28]; Kainz et al. [29]; Ragg [30]; Atreyapurapu et al. [31]; Barulina et al. [32]; Krishnan et al. [33]; Krishnan et al. [34]; Zolotukhin et al. [35]), as presented in Table 1. These studies were written by authors from different countries, including Canada, China, Germany, India, Japan, Kazakhstan, Russia, and the United Kingdom (UK), this consideration reflecting a world-wide interest in this topic.

The EBM levels and grades of recommendation of the included papers are presented in Table 2.

Considering the studies focus, the analyzed papers encompassed a wide range of topics related to AI involvement in venous pathology diagnosis, including clinical setting CVD images analyses in Shi Q et al. [27], Barulina et al. [32], and Zolotukhin et al. [35]; thermal images dataset analyses in Levshinskii et al. [28], Krishnan et al. [33], and Krishnan et al. [34]; ultrasound images in Kainz et al. [29] and Ragg [30]; and magnetic resonance venograms analyses in Atreyapurapu et al. [31].

### 3.2. Patient Populations and Study Volumes

A total of nine major studies were reviewed, encompassing 35,869 medical images, provided from 1169 patients. The largest dataset acquired 18,111 ultrasound records focused on epifascial leg-vein valve analysis [30], while smaller studies focused on pilot implementations [27] and algorithm validation [28,29]. The largest number of cases prospectively analyzed was 433 patients [35].

The patient age across these studies showed a broad range but typically reflected the more common age demographic affected by CVD [36,37], with mean ages from 40.8 ± 12.5 years in Krishnan et al. [34] to 46.0 years in Zolotukhin et al. [35]. However, age range varied among studies, the youngest patient enrolled being 15 years old and being noted in Krishnan et al. [34], and the oldest patient being 84 years old and being noted in Kainz et al. [29]. Gender distribution varied slightly among studies, with female predominance noted in most studies as common in CVD patients [38,39]; female gender distribution varied between 41.2% in Krishnan et al. [34] and 76.7% in Zolotukhin et al. [35].

Patients’ clinical data included a mean body mass index of 27.75 kg/m^2^ and a wide range of clinical presentations, from early stages of chronic venous disease [35] to advanced ones [32,34], and even acute complications like thrombosis [29]. The widest distribution of cases, according to the clinical–etiology–anatomy–pathophysiology (CEAP) classification, was noted in the study provided by Barulina et al., in which the distribution of images by CEAP classes was C0—7.83% (n = 872), C1—25.27% (n = 2810), C2—13.42% (n = 1493), C3—33.66% (n = 3743), C4—13.76% (n = 1530), C5—3.62% (n = 403), C6—2.4% (n = 267) [31], followed by a similar study group described by Zolotukhin et al., in which CVD class was noted as follows: C0(s)—30.5% (n = 132), C1—29.8% (n = 129), C2—21.2% (n = 92), C3-6—9.7% (n = 42), abnormalities not associated with—8.8% (n = 38) [35].

### 3.3. Artificial Intelligence Impact on Venous Pathology Diagnosis

According to the analyzed papers, AI proved to be a useful instrument in different stages of CVD diagnosis, including the patient’s first presentation and clinical setting assessment [27,32,35], ultrasound scan results [29,30], thermal images [29,33,34], and even magnetic resonance venogram analyses [30]. AI algorithms consistently achieved sensitivity and specificity exceeding 90% across multiple studies. In the study conducted by Shi Q et al., AI (a neural network) demonstrated superior performance and efficiency for CVD classifiers, with classification accuracy up to a 90.92%, kappa coefficient (a statistical measure that evaluates the agreement between raters by factoring out expected agreement due to chance) of 0.8735 and an F1 score (a measure of predictive performance) of 0.9006, outperforming doctors’ diagnoses, with accuracy, kappa, and F1 score improved by 9.11%, 0.1250, and 0.0955, respectively [27].

Levshinskii et al. analyzed the possibility of using artificial intelligence passive microwave radiometry for the diagnostics of venous diseases. The model described in this study achieved sensitivity of 0.812 and specificity of 0.729 [28].

The deep learning algorithm used for detecting vein thrombosis in the study conducted by Kainz et al. exhibited a diagnostic performance with sensitivity ranging between 0.82 and 0.94 (95% CI), specificity between 0.70 and 0.82, a positive predictive value between 0.65 and 0.89, and a negative predictive value between 0.99 and 1.00, when compared to the clinical standard. Additionally, the authors estimated that this algorithm might generate a positive net monetary benefit of up to £72 to £175 per software-supported examination [29].

In the study conducted by Ragg, a new AI program (A.I.M., VeinBrain, Feusisberg, Switzerland) was used to develop investigator-independent criteria for congenital or acquired vein valve lesions. AI proved useful, the individual origin of leg vein insufficiency being determined in 95.4% of cases involving patients 20 years old or younger, 82.6% in patients with ages between 21 and 40 years, and 74.1% in patients with ages between 41 and 60 years. However, AI was unable to determine the underlying causes and mechanisms of deterioration in 69.8% of patients over the age of 60, due to the presence of multiple overlapping factors (embryonal, pressure-induced valve decompensation, or stasis-related valve degeneration lesions) with uniform changes in morphology and function (valve degression) in late stages [30].

The study conducted by Atreyapurapu et al. focused on developing an artificial intelligence model capable of distinguishing between advanced CVI and early-stage CVI or normal legs while objectively quantifying soft tissue changes. A total of 5200 images obtained from magnetic resonance venograms were categorized as normal or abnormal and utilized for training, validation, and testing of the convolutional neural network model. The model achieved an accuracy of 97% in differentiating between normal and abnormal images [31].

The aim of the work conducted by Barulina et al. was to implement deep learning (DL) techniques for automatic classification of CVD stages for patient self-diagnosis by using images of the patients’ legs. Images of legs affected by CVD, necessary for deep learning (DL) algorithms, were gathered from publicly available online sources using the developed algorithms. For image preprocessing, a binary classification task distinguishing “legs vs. no legs” was performed using ResNet50, achieving an accuracy of 0.998. To classify different stages of CVD based on the CEAP classification, a multiclassification approach was employed using four neural networks with distinct architectures: ResNet50 and transformer-based models, including data-efficient image transformers (DeiT) and two versions of a custom vision transformer (ViT-base-patch16-224 and ViT-base-patch16-384). Among them, the DeiT model, even without fine tuning, outperformed the ResNet50 model, achieving a precision of 0.770 compared to 0.615 for ResNet50. The highest precision (0.79) was recorded with the ViT-base-patch16-384 model. To showcase the study’s findings, a fully functional deep learning algorithm was integrated into a Telegram bot, enabling the assessment of patients’ leg conditions with relatively high accuracy in CVD classification. Overall, the study reported a sensitivity of 94.7% and a specificity of 96.3% for detection of CVD complications using convolutional neural networks (CNNs) [32].

Krishnan et al. assessed the feasibility of using infrared thermographic image analysis supported by deep learning (DL) techniques in CVD early prediction and diagnosis. In this research, DenseNet-121 and other pre-trained convolutional neural network models, including EfficientNetB0 and Inception_v3, were trained using a transfer learning strategy. The experimental findings indicate that the proposed modified DenseNet-121 model outperformed other classical methods. The reported results provide evidence of the robustness of the suggested method in addition to the high accuracy that it achieved, as shown by the overall testing accuracy of 97.4% [32]. Also, the same collective of authors presented a lightweight DL-based convolutional neural network (CVINet) model which can identify healthy and CVD-affected subjects more precisely using infrared thermal images. The results have demonstrated that this method achieved a high classification accuracy of 96.8% on the thermal image dataset, with an initial learning rate of 0.0001 [34].

The study published by Zolotukhin et al. assessed the accuracy of the AIVARIX application in identifying C1–C2 CVD patients via analysis of images of their lower extremities. For class C1, the app showed sensitivity and specificity of 75.2% (95% CI 66.8–82.4) and 86.5% (95% CI 82.2–90.1), respectively; for class C2, the results were 93.5% (95% CI 86.3–97.6) and 82.7% (95% CI 78.3–86.6), respectively [35].

## 4. Discussion

According to the literature data, AI involvement in venous disease diagnosis has become more and more common in recent years, with different approaches being explored. For example, deep learning models were used as user-friendly and cost-effective screening tools for early diagnosis and monitoring of varicose veins. A custom deep learning model was trained on a meticulously curated dataset of varicose vein images, categorizing them as “Normal” and “Varicose”. The system also extends to real-time varicose vein detection through laptop cameras, providing instant visual feedback for timely intervention. This real-time capability is complemented by long-term monitoring, making it a valuable tool for both clinical and home-based use [40]. This approach aligns with modern healthcare requirements, enabling early intervention in varicose vein management and contributing to improved healthcare outcomes, this tool being easy to access for patients from different areas, even before they are seen by a physician. Especially for rural residents, AI-supported diagnostics seems to be a good option [41]. Also, a study addressed to phlebologists, which aims to evaluate the sensitivity and specificity of Ivenus application for determining the clinical class of chronic venous diseases (within C0-C2 according to CEAP), is ongoing [42].

Also, publicly available chat-bots brought promising results in assisting patients in home-based CVD diagnosis [43]. However, despite AI chat-bots like ChatGPT 3.5 and ChatGPT 4.0 demonstrating potential to provide clinically relevant answers in different tested scenarios, their reasoning must still be carefully analyzed for exactitude and clinical validity. In the experimental study conducted by Alexiou et al., the authors evaluated the performance of the GPT-4 AI model across 57 clinical cases sourced from a vascular surgery textbook. Custom prompts were designed to engage the AI model, requiring it to recognize symptoms, establish diagnoses, and recommend appropriate treatments. The model accurately responded to over 65% of the 385 questions. However, an analysis of the questions where accuracy dropped below 50% revealed limitations in interpreting and processing complex medical information, primarily due to difficulties in understanding intricate clinical scenarios. Additionally, the AI model provided incorrect or outdated information in 14% of cases and struggled with context, nuances, and medical classification systems in 11% of instances [44]. The literature data provide insights into machine learning models’ involvement in the diagnosis of cardiovascular diseases using easily accessible blood routine and biochemical detection data [45]. Considering the potential role of blood tests in diagnosing and monitoring CVD progression, these approaches suggest another further applicability of AI in venous disease diagnosis [39,46].

Of course, most of the literature data are related to AI usage in medical imaging assessment and generating radiological diagnosis. Many published papers are related to AI usage in diagnostic ultrasonography [47], and most of those which are related to vascular medicine are debating arterial assessment, including carotid artery stenosis and aortic and peripheral artery diseases [48]. To our knowledge and based on our findings, no comprehensive study has examined AI specifically used in lower-limb venous reflux assessment, but some insights in this regard were noted. For example, the study conducted by Lippi et al. describes potential applications of AI in vein recognition [49], which is the first important step for AI in order to perform further assessments and identify pathological aspects such as reflux. However, although the use of DL methods with ultrasound imaging in medical imaging has recently gained attention, it should be considered that its application in preclinical in vivo studies is still in its early stages [50].

Additionally to diagnosis, there are sources that provide information about AI implementation in venous disease treatment, complications, and follow-up assessments. AI algorithms have the capability to enhance patient outcomes by delivering more precise diagnoses, tailoring treatment plans to individual needs, and offering real-time assistance during minimally invasive procedures [51]. Through patient clinical data analysis and by considering their individual characteristics, such as age and overall health status, AI algorithms can suggest optimal treatment modalities. This personalized approach can enable healthcare professionals to develop treatment plans that address the unique needs of each patient, leading to improved outcomes and greater patient satisfaction [52]. Also, AI may aid in the diagnosis and prediction of venous thrombosis, demonstrating high sensitivity and specificity [53], and also play a role in venous thromboembolism prevention and management [54], thus bringing an important clinical benefit. Other promising areas for AI are varicose vein recurrence risk estimation after invasive procedures and predicting the healing time for venous leg ulcers [41].

The literature data provide information regarding applications of AI in other fields related to phlebology, like differential diagnosis of lymphedema and lipedema [55] and the management of esophageal varices [56].

In spite of the great potential of AI tools in improving venous care, an important issue which should be addressed is medico-legal liability. The controversy around legal liability when results are validated by AI remains a hot topic nowadays, because no specific regulations have been issued to date. However, more and more sources suggest that this aspect will be regulated in the near future, and AI tools will be able to be used in everyday practice, according to the issued legal regulations [57].

Overall, to date, the AI algorithms consistently achieved accuracy exceeding 90%; reduced inter-observer variability, ensuring consistent interpretation of ultrasonographic images across clinicians and settings; accelerated diagnostic workflows by reducing the time required for image analysis by over 50%; and demonstrated the ability to identify subtle abnormalities, such as minor venous reflux or early-stage thrombi, that might be missed during manual evaluations. Thus, the future of AI in venous diagnostics is promising. According to our literature research, several areas of development were noted, including AI algorithms embedding directly into ultrasound devices to provide instantaneous diagnostic insights during patient evaluations [58,59]; combining AI-processed Doppler data with other imaging modalities, such as computed tomography or MRI, for comprehensive assessments [31]; AI usage in order to predict disease progression and tailor treatment strategies based on individual patient profiles [41]; and constructing large-scale, multicenter datasets to improve the robustness and generalizability of AI algorithms [32,60,61]. By addressing these directions, AI is expected to revolutionize venous care, transitioning from a supplementary tool to an integral component of clinical practice. The potential value of AI technology in future clinical practices consists in direct integration of AI-enabled tools into ultrasound devices, multimodal data analysis, and the development of personalized treatment strategies.

Another consideration which should be discussed is the introduction and widespread application of AI systems in CVD screening. The development of a system that identifies patients presenting with minor clinical symptoms suggestive of venous disease and integrates them into a centralized database for CVD risk is becoming a reality. This process considers family and pathological history (including surgeries and genetic risk factors [40]), lifestyle habits, specific symptoms frequency, and evolving imaging. Based on an integrated system and specific classifications, an adequate selection of patients at risk of developing CVD can be made and specific prevention protocols can be developed, both by implementing medical conditions and by modifying unhealthy lifestyle habits. Considering that it is easier to prevent than to treat, the integration and application of AI systems in CVD screening and prevention may become a future standard.

Considering the RQs addressed in this paper, according to the data analyzed and presented in this manuscript, we can state the following: the current literature captures a wide range of topics related to AI involvement in venous pathology diagnosis, from clinical-setting CVD image analysis to thermal or ultrasound image and magnetic resonance venogram analyses (RQ1); AI algorithms have consistently provided accurate results, exceeding 90% in most of the published studies (RQ2); according to the recent literature, AI is expected to revolutionize venous care, and most likely in the near future it will become a common tool in daily practice adopted by most phlebologists.

While this review paper provides a valuable perspective and overview of the AI-enabled tools used in phlebology, some limitations of this study should be discussed. The sample size varies among the studies, and different kinds of algorithms are described, which may limit the generalizability of the results. One of the RQs addressed in this review was to assess the accuracy of AI-enabled tools used in phlebology. While the efficacy of the AI models seems to be high, the results come from different AI model assessments, AI-enabled tools not being currently used in clinical practice at the moment. Multicentric prospective studies are required for a better understanding of AI-enabled tools’ impact in phlebological daily practice. In order to enhance the robustness and generalizability of AI algorithms, large-scale, multicenter databases should be further considered.

## 5. Conclusions

Artificial intelligence represents a transformative innovation in the diagnosis and management of venous diseases. By enhancing diagnostic accuracy, streamlining workflows, and enabling personalized care, AI has the potential to address current challenges in venous diagnostics and improve patient outcomes. One of the most compelling findings of this review is AI’s capacity to detect subtle abnormalities that may be overlooked during manual evaluations, such as early-stage thrombi or minor venous reflux. This underscores the potential of AI not only as a diagnostic tool but also as a means to enhance preventive strategies by identifying at-risk patients earlier in the disease progression. Advancements in AI-driven deep learning models, including CNNs and transformer-based architectures, have demonstrated superior classification performance in their ability to distinguish between different stages of venous disease, offering precise and reproducible results.

Another considerable role of AI is in venous disease screening and prevention. By utilizing AI to incorporate patient history, lifestyle factors, and imaging data into centralized databases, clinicians could more effectively identify individuals at high risk of CVD. This would allow early interventions, lifestyle modifications, and treatment plans aimed at preventing disease progression, rather than solely managing the later-stage complications.

As research progresses, the integration of AI into routine clinical practice will likely become a standard, marking a new era in phlebology.

## Figures and Tables

**Table 1:** Characteristics of studies evaluating AI involvement in venous pathology diagnosis.

Study	Author’s Country of Affiliation	Year	Study Design	Topic
Shi Q et al. [27]	China	2018	Observational	Automatic classification method for chronic venous insufficiency images
Levshinskii et al. [28]	Russia, UK, Japan	2021	Prospective	AI and passive medical radiometry usage for venous disease diagnostics
Kainz et al. [29]	UK, Germany, Canada	2021	Prospective	Machine learning usage in ultrasound deep vein thrombosis diagnosis.
Ragg [30]	Germany	2021	Observational	AI involvement in venous insufficiency assessment
Atreyapurapu et al. [31]	India	2022	Observational	Assessment of anatomical changes in advanced chronic venous insufficiency by using AI and machine learning techniques.
Barulina et al. [32]	Russia, Kazakhstan	2022	Prospective	Deep learning approaches to automatic chronic venous disease classification
Krishnan et al. [33]	India	2023	Observational	Chronic venous disease diagnosis by using transfer learning with convolutional neural networks based on thermal images
Krishnan et al. [34]	India	2024	Prospective	A deep learning based model for the diagnosis of chronic venous insufficiency in lower extremity using infrared thermal images
Zolotukhin et al. [35]	Russia	2024	Prospective	AI-based application accuracy in early stages of chronic venous disease diagnostics

**Table 2:** EBM level and the grade of recommendation of the included papers. (EBM Classification Explanation: Level I: Randomized controlled trials (RCTs) or systematic reviews of RCTs; Level II: Well-designed prospective studies, cohort studies; Level III: Case-control studies, observational studies; Level IV: Case reports, expert opinion, or series. Grade of Recommendation: Grade A: Strong evidence from Level I studies; Grade B: Moderate evidence from Level II–III studies; Grade C: Weak evidence from Level IV or conflicting results).

Study	EBM Level	Grade of Recommendation
Shi Q et al. [27]	Level III	Grade B
Levshinskii et al. [28]	Level II	Grade B
Kainz et al. [29]	Level II	Grade B
Ragg [30]	Level III	Grade B
Atreyapurapu et al. [31]	Level III	Grade B
Barulina et al. [32]	Level II	Grade B
Krishnan et al. [33]	Level III	Grade B
Krishnan et al. [34]	Level II	Grade B
Zolotukhin et al. [35]	Level II	Grade B

## Data Availability

No new data were created or analyzed in this study.
